# The Many Questions about Mini Chromosomes in *Colletotrichum* spp.

**DOI:** 10.3390/plants9050641

**Published:** 2020-05-19

**Authors:** Peter-Louis Plaumann, Christian Koch

**Affiliations:** Division of Biochemistry, Department of Biology, Friedrich-Alexander-Universität Erlangen-Nürnberg, 91058 Erlangen, Germany; peter-louis.plaumann@fau.de

**Keywords:** mini chromosome, dispensable chromosome, virulence chromosome, B-chromosome, lineage-specific region, accessory region, effector, plant pathogens

## Abstract

Many fungal pathogens carry accessory regions in their genome, which are not required for vegetative fitness. Often, although not always, these regions occur as relatively small chromosomes in different species. Such mini chromosomes appear to be a typical feature of many filamentous plant pathogens. Since these regions often carry genes coding for effectors or toxin-producing enzymes, they may be directly related to virulence of the respective pathogen. In this review, we outline the situation of small accessory chromosomes in the genus *Colletotrichum,* which accounts for ecologically important plant diseases. We summarize which species carry accessory chromosomes, their gene content, and chromosomal makeup. We discuss the large variation in size and number even between different isolates of the same species, their potential roles in host range, and possible mechanisms for intra- and interspecies exchange of these interesting genetic elements.

## 1. Introduction

Originally described as B-chromosomes in the bug *Metapodius* by Wilson in 1907, remarkably small chromosomes were first identified in fungi 30 years ago [1]. Since then, different names have been used to describe them [2]. ”Supernumerary chromosomes”, “accessory chromosomes”, “conditionally dispensable chromosomes”, “lineage-specific chromosomes”, or simply “mini chromosomes”—all of these terms have been used to describe some of the striking features. Next-generation DNA sequencing revealed that they tend to differ from the rest of the chromosomes (core or A chromosomes) in size, gene density, transposable element (TE) content, sequence variability, and (GC) content. In this review, we will call them “accessory chromosomes” or “mini chromosomes” because they are usually much smaller (<2 mb) than their respective core chromosomes. In one case, however, a dispensable chromosome as large as 3 mb was referred to as a ‘mini’ chromosome [3]. For some fungal species, genome sequencing identified so-called lineage-specific regions (LS regions) within the core genome with sequence features comparable to mini chromosomes, and which clearly differ from the rest of the genome. We will refer to these as “accessory regions”. 

A few years ago, Dong, Raffaele, and Kamoun suggested the concept of a two-speed genome of filamentous plant pathogens [4]. The basic statement is clear and straightforward [4,5,6,7]. The core genome includes widely conserved genes important for vegetative physiology, i.e., housekeeping genes, and is defined as a compartment that is genetically stable, largely under negative selection and, consequently, does not evolve fast. The second part of the genome evolves much faster and tends to be highly enriched with transposable elements (TE) and other repetitive elements, which are thought to promote diversification of the genome. The latter description also fits the characteristics of accessory chromosomes or regions. The presence of accessory chromosomes encoding virulence-related genes may be the result of such a split genome. The situation is somewhat similar to the genomic organization of bacteria, despite the known differences in genomic plasticity: Apart from the core genome, conjugative plasmids, which confer beneficial functions under certain conditions, are mobile, and usually enriched with insertion sequence (IS) elements and composite transposons. The tumor inducing (Ti) plasmid of *Agrobacterium tumefaciens* may be such an example.

Accessory chromosomes were found in diverse lineages of plant-pathogenic filamentous ascomycetes (Table 1). In obligate biotrophic pathogens, such as *Blumeria graminis,* accessory chromosomes or accessory genetic regions have not been described. Genome sequencing of two different *formae speciales* of powdery mildew (*B. graminis f.* sp. *tritici* and *B. graminis f.* sp. *hordei*) revealed a more or less even distribution of transposable elements and virulence factors within the whole genome [8,9]. For hemibiotrophic and necrotrophic fungal plant pathogens, however, accessory chromosomes and accessory genetic regions might be a common feature, as we will show. Moreover, different species from the *Pythium* genus of filamentous oomycete pathogens also harbor one or two accessory chromosomes [10]. Whether they contribute to virulence or not has never been reported. In this review, we outline the situation of mini chromosomes and accessory genetic regions in *Colletotrichum* and related pathogens.

## 2. Mini Chromosomes in *Colletotrichum* and Other Plant-Pathogenic Fungi

### 2.1. Colletotrichum gloeosporioides

Accessory mini chromosomes in *Colletotrichum* have first been described in *Colletotrichum gloeosporioides* in 1990 [11] when Pulsed-Field Gel Electrophoresis (PFGE) had been introduced [12], which allowed for electrophoretic karyotyping. Large variations in size and number of accessory chromosomes (up to 10) in different isolates of the same species were observed. In a subsequent study, Masel and colleagues identified chromosomal variations between strains, which are pathogenic for different cultivars of the host *Stylosanthes* spp. [13]. This suggested a role of these accessory regions in host adaptation or general virulence. However, no detailed information concerning the gene content of any chromosome was available at that time, so direct evidence for virulence factors encoded on these chromosomes was lacking. A partial sequence of a mini chromosome was deposited at the National Center for Biotechnology Information (NCBI) (Nourse et al. unpublished, Accession AF448489.1).

Interestingly, the transfer of such an accessory mini chromosome between two otherwise vegetatively incompatible isolates (A- and B-type) of *C. gloeosporioides* was observed at low frequencies in vitro when cell fusion was forced using a selectable marker integrated in the mini chromosome [14]. Although the transferred chromosome was required for virulence on a certain host in the donor strain, it was not sufficient to allow a host shift in the receiving strain. Nothing is known about the transfer mechanism and how mechanisms for allorecognition could be overcome [15]. Nevertheless, such events may have contributed to the evolution of this pathogen [14]. 

These studies date back 20 to 30 years. Since then, many more members of the *gloeosporioides* species complex have been recognized and described (in total 22 and one subspecies) [16]. Nowadays, commercially important pathogens are assigned to this species complex, including, for example, *Colletotrichum fruticola* and many more. Although several of these species or isolates of the same species have been sequenced in recent years, full genome assemblies are only slowly emerging [17]. 

Given the large number of related *Colletotrichum* species, the question arises whether all species/races and strains carry mini chromosomes or not. As for *Colletotrichum orbiculare,* no mini chromosomes were detected using a cytologic approach, nor by genome analysis [18,19]; the most likely answer will be “no”. The absence of mini chromosomes, however, does not exclude the possibility that regions with similar functions exist integrated into the core genome as accessory regions with similar characteristics generated either by duplication or by transposition.

### 2.2. Colletotrichum higginsianum and Colletotrichum graminicola

*C. higginsianum* (isolate IMI349063) and *C. graminicola* (isolate M1.001) were the first two *Colletotrichum* species that were sequenced using 454 and Illumina sequencing [22]. Combined with optical mapping, this revealed the presence of two and three accessory chromosomes in *C. higginsianum* and *C. graminicola*, respectively. Comparative analysis, however, did not show any synteny between the mini chromosomes of the two species. It was further noted that the sequences of mini chromosomes from two different sexually compatible *C. graminicola* strains (M1.001 and M5.001) differ, suggesting variations in this species [41]. Taga and colleagues validated the presence and number of mini chromosomes using a cytologic approach [18]. 

**Figure 1:**
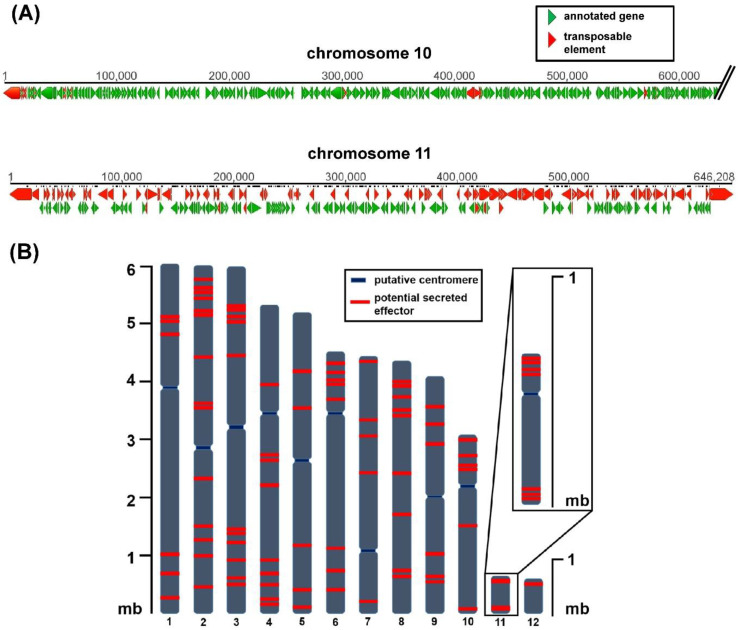
Representation of the genome structure and effector candidate distribution on chromosomes of *C. higginsianum* (**A**) Comparison of sequences from core chromosome 10 and mini chromosome 11. Red bars display transposable elements, green bars display annotated genes. The first 600 kb of chromosome 10 are shown here. Software Geneious 5.5.9 (**B**) Distribution of effector candidate genes (red bars) on chromosomes of *C. higginsianum*. *n* = 87, annotated as *C. higginsianum* effector candidate genes (ChECs) in [20].

*C. higginsianum* was re-sequenced using single molecule real-time (SMRT) techniques [42] which led to the first gapless genome assembly for all 12 chromosomes, 10 core chromosomes of 3–6 mb, and two mini chromosomes (chromosome 11 approximately 650 kb, chromosome 12 approximately 600 kb) [20]. The complete genome assembly allowed the genomic location and distribution of potential virulence determinants to be analyzed. Both mini chromosomes were enriched with putative effector genes and genes with unknown function while the total amount of protein-coding genes was much lower than for the core chromosomes [20]. Almost 40% of mini chromosome 11 consists of transposable elements, which may be the reason for the observed reduced GC content. These differences between core and mini chromosomes are schematically shown in Figure 1A. Kleemann and colleagues have identified a large set of *C. higginsianum* effector candidate genes (ChECs) [43,44]. When their distribution across the genome is analyzed, they are not only frequently found on mini chromosomes, but those located on core chromosomes also appear to be non-randomly distributed (Figure 1B). On average, around 80% of all effector candidates are either located within the first or the last quarter of a chromosome. Such a distribution of potential effector genes is reminiscent of accessory regions close to telomeres in *Magnaporthe* [3,45] and potentially *Fusarium* [46].

For the *C. higginsianum* isolate MAFF305635, it was shown that mini chromosome 11 was crucial for virulence on the model plant *Arabidopsis thaliana*, but dispensable for vegetative growth [21]. Mutants lacking mini chromosome 11 were still able to penetrate host cells but became arrested during the biotrophic phase of infection, suggesting that chromosome 11 harbors genes specifically involved in suppressing plant defense mechanisms. Indeed, *C. higginsianum* mutants lacking chromosome 11 were shown to successfully infect *A. thaliana* mutants lacking critical immune response genes. In particular, *Arabidopsis* mutants lacking tryptophan-derived secondary metabolites (*cyp79 b2/cyp79 b3* double mutants) were susceptible to *C. higginsianum* mutants lacking mini chromosome 11 [21]. In contrast, mini chromosome 12 was dispensable for both virulence on *A. thaliana* and saprophytic growth. Whether or not chromosome 12 has a role in virulence on other hosts is not known.

The available genomic sequence data for two *C. higginsianum* strains allowed sequence variations within mini chromosomes to be analyzed. The frequency of nucleotide exchanges between *C. higginsianum* isolates IMI349063 [20] and MAFF305635 [21] suggested that coding sequences of these two isolates from chromosome 11 are more similar to each other than coding sequences from the core genome (0.12 single-nucleotide polymorphisms (SNPs)/kb compared to 1 SNPs/kb). Coding sequences from chromosome 12, however, appeared to be less conserved between those two isolates (5.27 SNPs/kb compared to 1 SNPs/kb) [21]. Moreover, both mini chromosomes show size variations in the two *C. higginsianum* isolates (IMI349063: 648 kb and 598 kb; MAFF305635: ~620 kb and 800 kb). Size variation in mini chromosomes may be a common feature of these elements as such variations have been observed in several instances [11,26,27,47]. The many transposable elements on mini chromosomes (chr11: 38.4% and chr12: 28% of all sequences) may be responsible for these apparently fast changes in size [20] and possibly also for an altered gene order (Figure 1). This clearly supports the assumption of a fast-evolving genomic compartment, which might drive the adaptation of *C. higginsianum* to its environment or hosts [48].

### 2.3. Colletotrichum lentis

Mini chromosomes have also been identified in the lentil (*Lens culinaris*) pathogen *Colletotrichum lentis* [23]. *C. lentis* has been assigned to the *Colletotrichum destructivum* clade [49] and is phylogenetically close to *C. higginsianum* [50]. The genome size of 56 mb is similar and *C. lentis* also harbors 12 chromosomes two of which are referred to as mini chromosomes [23]. This study showed that a genomic region from a mini chromosome carries genes important for virulence. After crossing two compatible strains with differences in virulence, the chromosomal DNAs of many meiotic segregants were sequenced to generate a linkage map of SNPs. The genetic linkage allowed the verification of the genome assembly (N_50_ scaffolds of 4.9 mb) and supported the presence of 12 chromosomes despite the lack of telomeric sequence reads for all of them. The two strains belonged to the originally described race 0 (CT-30) and the less virulent race 1 (CT-21). The strains exhibit strong quantitative differences in virulence and can be crossed. This not only allowed genetic fine mapping but also identified a region on mini chromosome 11 that carries a quantitative trait locus (QTL) largely responsible for the difference in virulence between the two races. Therefore, *C. lentis* harbors a mini chromosome critically important for virulence, just like *C. higginsianum* [21]. Significantly, when this genomic region is compared to the *C. higginsianum* genome, 22 genes from the QTL have homologs in *C. higginsianum.* Six of these 22 genes have homologs on mini chromosome 12 in *C. higginsianum*. However, none of the genes from this QTL is related to genes on virulence chromosome 11 in *C. higginsianum*. Which gene or genes from the QTL region are critical for virulence is not yet known. The region encompassing the QTL in *C. lentis* encodes the genes PDA1 and pep1 with similarity to pathogenicity genes found in a virulence gene cluster on a mini chromosome in *Fusarium solani* [51,52]. The mini chromosomes in *C. lentis* share their low gene content and high TE content with mini chromosomes from *C. higginsianum*. The mitotic stability of mini chromosome 11 in *C. lentis* has not been tested, but meiotic instability was not observed in crosses [23]. Interestingly, crossing over was observed for both *C. lentis* mini chromosomes in genetic crosses, indicating that meiotic recombination may occur at rates comparable to the core genome.

### 2.4. Other Colletotrichum Species

To date, more than 100 *Colletotrichum* species have been described [40]. In addition, an unknown number of different isolates from different areas and hosts have been collected for many species. For this genus, 11 species complexes have been described and accepted largely based on sequences from ITS and a limited number of marker genes [50,53]. Three additional species complexes were recently proposed [54]. The number of reported genome sequences is increasing steadily [50], which will eventually enable the detailed comparison of accessory regions and mini chromosomes.

A possible genetic association between mini chromosomes and virulence was observed in *Colletotrichum kahawae*, the causal agent of the devastating coffee berry disease (CBD) causing up to 80% yield loss annually [55]. Phylogenetic studies suggested that *C. kahawae* recently emerged from the *gloeosporioides* species complex by gaining the ability to infect green coffee berries [55]. Using PFGE analysis, Pires and colleagues [26] identified mini chromosomes and found a correlation between virulence and the presence or number of mini chromosomes in different *C. kahawae* isolates. However, there was no correlation detected between virulence and genome size, which was estimated using a flow cytometry approach. The authors reported that, independent of the geographical origin of different strains, medium- and high-aggressive isolates carried four to five mini chromosomes each. Low-aggressive isolates carried only two to three. In addition, the three most aggressive isolates all carried one mini chromosome of the same size (700 kb). Two isolates with a significantly different level of aggressiveness shared almost the same karyotype except for the presence of a mini chromosome. Despite the lack of sequence information, this study showed how much variation there can be within this species complex in number and size of mini chromosomes. These findings also suggested important roles of mini chromosomes in virulence of *C. kahawae*. More recently, a genome-wide association study using restriction site-assisted DNA sequencing (RADseq) for SNP calling was conducted [56]. The comparison of 30 *C. kahawae* isolates from 10 African countries and 10 non-pathogenic isolates from different host plants across the world identified four candidate genes possibly involved in signaling, detoxification, and gene expression, which might facilitate the specialization and adaptation to its host.

The causal agent of the black spot disease on common beans (*Phaseolus vulgaris*), *Colletotrichum lindemuthianum*, belongs to the clade of *orbiculare* and harbors accessory mini chromosomes [27]. Using electrophoretic karyotyping, the presence of two to six chromosomal bands smaller than 2.5 mb was observed in different isolates. Based on southern blot analysis, these were shown to be rich in repeated sequences. In addition, sequences present on a mini chromosome were completely lost from the genome together with the mini chromosome, suggesting the loss of a whole, dispensable chromosome rather than translocation of sequences to the core genome.

A similar situation was observed in *Colletotrichum acutatum,* which, eponymous for the *acutatum* clade, can infect many different plants including strawberries [57]. Garrido and colleagues showed that the estimated genome size of different isolates from distinct geographical sites ranged from 30 to 37.5 mb [24]. The total chromosome number in those strains was between six and nine and one or two mini chromosomes smaller than 1 mb were found. A more recent study (strain KC05 from South Korea) revealed a genome size of approximately 52 mb, which is more similar to many other *Colletotrichum* species [58]. Comparative genomics of four members of the *acutatum* clade suggested that lineage-specific expansions, i.e., duplications, as well as lineage-specific losses, led to the specialization and adaptation to specific hosts [59].

Collectively, these findings indicate that accessory chromosomes may, in fact, be a common feature in the *Colletotrichum* genus. There are, however, also exceptions. As mentioned before, *C. orbiculare* was reported to lack mini chromosomes [18]. Instead, the genome analysis revealed a large genome expansion involving repeated elements leading to a two-fold larger genome size compared to *C. gloeosporioides.* [60]. It would be interesting to see whether these regions have roles in virulence or not. The absence of mini chromosomes need not be the case for all *C. orbiculare* isolates. As studies with *C. gloeosporioides* [25] or *C. kahawae* [26] revealed, there is a high variability of accessory chromosomes in different isolates within the same species. The same might be true for *C. orbiculare* isolates. Gan and colleagues [19] reported the genome assemblies of four members of the *C. orbiculare* species complex (*C. orbiculare*, *C. trifolii*, *C. sidae,* and *C. spinosum*). Although there is no telomere-to-telomere assembly available, the authors reported AT-rich and gene-poor regions, which may represent accessory genetic regions.

*Colletotrichum sublineola*, the causal agent of the anthracnose disease in sorghum (*Sorghum bicolor*), belongs to the species complex of *graminicola* and is closely related to *C. graminicola* [61]. Comparative genomic studies with *C. graminicola* did not provide evidence for mini chromosomes in *C. sublineola*. However, as far as we know, no other methods like electrophoretic karyotyping or optical mapping were used to analyze this. Considering the close relationship of the two organisms, it may be possible that mini chromosomes will eventually be found in *C. sublineola*.

In addition, it is conceivable that mini chromosomes are lost during propagation in the laboratory or in culture collections, as mini chromosomes can be genetically unstable [21]. This is not too far-fetched since a recently published genome of a transgenic *C. higginsianum* variant lacked mini chromosomes [62] previously identified [20,21].

### 2.5. Comparison to Other Plant-Pathogenic Fungi

There are several examples of accessory genomic regions and mini chromosomes in fungal pathogens, and often a correlation between the presence of these elements and virulence was observed (Table 1). In the early nineties, Miao and colleagues observed a small, meiotically unstable chromosome in *Nectria haematococca* (teleomorph of *Fusarium solani*). This chromosome was dispensable for normal growth but necessary for pathogenicity against its host pea [63]. The mini chromosome encodes for a gene that facilitates the detoxification of the antimicrobial phytoalexin pisatin. The genetic association of virulence with the mini chromosome was shown by inserting telomere-containing DNA fragments leading to mini chromosomes with terminal deletions and which were impaired in their virulence [46]. For the related species *Fusarium oxysporum*, it was shown that a pathogenicity chromosome can be transferred from one strain (Fol007—pathogenic on tomato) to another (Fo-47—non-pathogenic on tomato) by introducing antibiotic resistance genes into the genomes followed by simple co-incubation and selection. This allowed to convert the non-pathogenic isolate into a pathogen of tomato [28]. The authors coined the term lineage-specific (LS) chromosomes for these elements.

The causal agent of the black leg disease on different *brassica* plants, *Leptosphaeria maculans*, possesses a mini chromosome whose size varies between strains [36]. By crossing different isolates, Balesdent and colleagues [35] observed that a mini chromosome of approximately 1 mb contains an avirulence gene (AvrLm11) whose presence results in an immune response of the host if the infected plant carries the respective R-gene (RLm11). Very similar to *C. higginsianum*, the mini chromosome carries regions highly enriched with transposable elements and only few genes. They also observed that the mini chromosome is frequently lost during meiosis (in approximately 5% of meiotic segregants). Although mini chromosomes were lost frequently, the AvrLm11 frequency was stable over a ten-year period in tested field isolates [35]. Genetic crosses also demonstrated that recombination between mini chromosomes can occur during meiosis. This may also lead to mini chromosomes of different sizes [36].

There are different pathotypes of *Alternaria alternata* adapted to different host plants via the production of host-specific toxins [64]. Pathogenic isolates share a common mini chromosome smaller than 1.7 mb [33]. Genes for toxin-producing enzymes are encoded on these mini chromosomes, for instance in the strawberry pathotype [34]. For another *Alternaria* strain, an unusual dual host specificity was observed [65]. Here, genes on different mini chromosomes are responsible for the synthesis of two different toxins. This may be a hint pointing to an intra-species transfer of chromosomes between two otherwise genetically isolated pathotypes of *Alternaria alternata* [65].

For *Magnaporthe* pathogens, the situation concerning mini chromosomes is less obvious. The reference strain of the rice blast fungus *M. grisea* 70-15 [66] harbors seven chromosomes ranging from 3.42 mb to 8.32 mb but no mini chromosomes (Accession: GCA_000002495.2). However, a variable number of small chromosomes were found in different *M. grisea* field isolates [47]. This again demonstrates the high variability even between different isolates of the same species. In *M. grisea* strain 84R-62B, a 1.6 mb mini chromosome was identified and found to carry the avirulence gene AvrPik. Southern blot analysis suggested that this chromosome is a chimera of an accessory chromosome and core chromosome 1 [30]. In *Magnaporthe* species, it appears that sequences with a signature typical for mini chromosomes can also be found elsewhere in the genome. The *Magnaporthe oryzae* isolate Ina168 was shown to carry a 1.7 mb lineage-specific region on a core chromosome whose presence correlated with virulence of the strain [67]. Peng and colleagues identified one to two mini chromosomes of approx. 2 mb and a coding capacity for fewer than 200 proteins in the *M. oryzae* lineage causing wheat blast [3]. The mini chromosomes were enriched in transposable elements often found in subtelomeric regions of the core genome. When different field isolates were sequenced and compared, it was found that the mini chromosome(s) from reference strain B71 were absent in one strain (T25) and of different size in another (P3). The genes for two effectors (BAS1 and PWL2) that are encoded on core chromosomes in the rice pathovar MG 70-15, are present on mini chromosomes in strain B71, suggesting that these regions can be mobile.

*Zymoseptoria tritici* (formerly known as *Mycosphaerella graminicola*) is a severe pathogen on wheat and is responsible for huge yield losses worldwide [68,69]. *Z. tritici* possesses up to eight accessory (and dispensable) chromosomes [38]. This large number is quite unusual and, since meiosis occurs in this fungus, it allows the genetic and ecological fates of mini chromosomes to be followed [70]. Wittenberg and colleagues observed that these chromosomes are frequently lost during meiosis. In one instance, however, non-Mendelian segregation with supposedly additional replication of unpaired accessory chromosomes was suggested because the unpaired, i.e., monosomic, mini chromosomes can unexpectedly show up in all meiotic segregants of a cross [39]. During mitosis, mini chromosomes in *Z. tritici* are lost with very high frequency (up to 50%/generation) in vitro and in planta [37]. Resequencing of strains after mitotic chromosome loss revealed that chromosome breakage frequently occurred in subtelomeric regions leading to shorter versions of the progenitor chromosomes, which were likely healed by de novo generation of telomeres. Moreover, the loss of chromosomes was found to affect virulence [38].

*Botrytis cinerea* is recognized as one of the most severe fungal pathogens worldwide due to its necrotrophic lifestyle and broad host range [71]. Using PFGE, different *B. cinerea* isolates showed up to three mini chromosomes [32]. A recent study revealed two mini chromosomes with sizes of 247 and 209 kb, which encode for uncharacterized proteins [31]. So far, no association of mini chromosomes with virulence has been described for this pathogen.

*Verticillium dahliae* is closely related to the *Colletotrichum* genus, both belonging to the *Sordariomycetes*, but does not appear to carry any mini chromosome. There are, however, varying numbers of so-called lineage-specific regions in different strains, enriched with transposable elements, often gene-poor and with high variability and plasticity in comparison to the core genome. Moreover, they were enriched with pathogenicity factors whose presence was shown to be crucial for virulence [72]. Depotter and colleagues recently showed that those LS regions may be more conserved between each other than the rest of the genome [73].

## 3. Mechanisms and Outlook

We are learning more and more about the presence of accessory regions and mini chromosomes as more chromosome-level genome assemblies become available. Maybe the most important feature of these genetic regions is their reported roles in virulence. There are several interesting questions to be asked in the future: Why are effector genes abundant on those genetic elements? How did these regions arise and is there a continuous de novo mechanism for the generation of new or altered mini chromosomes? Is there exchange of these genetic elements within or possibly between species? Are mini chromosomes contributing to host adaptation or even speciation?

### 3.1. Is There an Intra-Species or Even an Inter-Species Exchange of These Genetic Elements?

This question might be the simplest one to answer, since for *Fusarium oxysporum f.* sp. *lycopersici* it was described that a lineage-specific chromosome is frequently transferred under laboratory conditions, converting a non-pathogenic isolate to a pathogen of tomato [28]. The transfer mechanism was not described but the simple co-incubation of donor and recipient strain was sufficient, although the strains are not thought to naturally undergo mating. In that study, it was also observed that chromosomal transfer appeared to be restricted to lineage-specific (LS) chromosomes. The transfer of parts of core chromosomes was observed in a more targeted approach [29]. For two *Colletotrichum* species, *C. lindemuthianum* and *C. gossypii*, Roca and colleagues [74] could show that conidial anastomosis tubes (CATs) can be formed between species in the lab. This resulted in fusions of *C. lindemuthianum* and *C. gossypii* with mixed genomes, without a phase of meiotic recombination [75]. In addition to such immediate exchanges, there are indications for horizontal gene transfer (HGT) between the clade of *Magnaporthales* and *Colletotrichum* [76]. Using a phylogenomic approach, Qiu and colleagues identified 93 genes, which were possibly transferred between *Magnaporthales* and *Colletotrichum* in the past. Interestingly, 29 of the 93 putative HGT-events are genes coding for carbohydrate-active enzymes (CAZymes) which are known to be involved in virulence in a lot of plant-pathogenic fungi. They also observed that transferred genes tend to be genetically linked, i.e., may have been transferred together. Maybe mini chromosomes have been involved in such transfers.

Mobility of LS chromosomes or mini chromosomes in populations can occur through mating and meiotic recombination. For most *Colletotrichum* species, however, mating has not been observed. In other cases, including *L. maculans,* meiotic instability of mini chromosomes has been observed [35]. Most studies covering this topic in fungal species were conducted using *Zymoseptoria tritici* as a model organism. Wittenberg and colleagues [70] observed that mini chromosomes were lost frequently in crossings of genetically unrelated parents due to nondisjunction during meiosis II and less frequently during meiosis I when homologs are separated. So-called distributive disjunction [77] in which two non-homologous chromosomes are separated during the first meiotic division has been proposed to operate in *Z. tritici* [70]. This may involve the pairing of transposable elements or other elements through limited homology. Such ectopic pairing may also be the source for *de novo* generation or rearrangements between mini chromosomes. 

### 3.2. How Did Accessory Regions and Mini Chromosomes Arise and Is There a Continuous De Novo Mechanism for the Generation of New or Altered Mini Chromosomes?

One possible mechanism for how mini chromosomes arose might be from core chromosomes. Croll and colleagues suggested such a mechanism for the mini chromosomes of *Zymoseptoria tritici* [78]. Non-allelic, ectopic recombination of homologous chromatids during meiosis can lead to chromosome recombinants, in which one product becomes acentric and is likely lost during anaphase while the other becomes dicentric. The presence of two centromeres may also initiate a breakage-fusion-bridge (BFB) cycle, as first described by Barbara McClintock [79]. Since one telomere is lacking after chromosome breakage, such fragments get smaller during each cell cycle until the chromosome is healed by the addition of a new telomere [80]. It is not too difficult to imagine how chromosome fragments can acquire the three essential components of a chromosome (centromere, telomeres and origins of replication). Origins of replication, which identify as simple origin recognition complex (ORC) binding sites [81], can be found across the whole genome. In yeast, their distance is between 30 and 60 kb on average [82]. Telomeres can quite easily be healed by recombination mechanisms as has been shown for yeast [83]. Centromeric regions contain many repetitive- and AT-rich sequences, which may be derived from transposable elements and are bound by centromeric histone H3 variants (CenH3) [84]. Consequently, it is easy to imagine how a piece of a broken chromosome, preferably rich in TEs, can be healed to become a normal but small chromosome. Such chromosome breaks can also occur during mitosis or by double-strand breaks not healed by non-homologous end joining (NHEJ). Maybe one of the reasons why mini chromosomes are small simply reflects the distribution of functional replication origins on chromosomal fragments. In vertebrates, chromosomal fragments not too dissimilar to mini chromosomes can be observed in tumors where a process called chromothripsis has been described to generate many chromosome breaks [85]. Such pieces of extrachromosomal DNA can be identified as paired chromosome fragments in metaphase and were therefore called double minutes [86].

Many observations suggest that both mini chromosomes and the accessory regions of the fast-evolving parts of fungal genomes are subject to duplications and/or translocations typically associated with the activity of transposable elements. For *Verticillium dahliae*, LS regions appear to be segmental duplications whose generations may involve transposition. It was suggested that LS regions carry evolutionarily young transposable elements. Using RNASeq, those TEs were found to be transcribed, so they may be active [87]. 

Similar conclusions about the genomic organization and LS regions were drawn for genomes of *Magnaporthe oryzae* isolates yet no transcriptional activity of TE elements was reported [88]. In *C. higginsianum*, Dallery and colleagues also reported segmental duplications associated with transposable elements [20]. They found six duplications in total of which one was an intra-chromosomal duplication on mini chromosome 11 containing the candidate effector gene ChEC7 and two other potentially secreted proteins. Moreover, four of the six duplications were in subtelomeric regions (within 30 kb of the telomeres) containing at least one copy of a telomere-associated TE, which may provide sites for ectopic homologous recombination. Partial copies of effector genes (e.g., EC12a) can be found in the *C. higginsianum* genome, which, at first glance, look similar to pseudogenes. It is not known whether mechanisms of RNA reintegration [89] are operating or not.

Tsushima and colleagues [62] showed for *C. higginsianum* isolate MAFF305635 that effector genes are in closer distance to TEs compared to randomly chosen genes. Moreover, the intergenic regions observed were different for effector candidates compared to randomly chosen genes. They also stated that the large-scale rearrangements seen in the genome of *C. higginsianum* MAFF305635 relative to IMI349063 (between chromosomes 4 and 10) can be associated with transposable elements.

While chromosomal breaks, transposition, non-reciprocal recombination and healing of ends generated by chromosome breaks, all provide possible mechanisms for the generation of mini chromosomes, an obviously important question concerns the time frame. How often do such events occur and how ’mobile‘ are genes on mini chromosomes? Two observations suggest that such events could be quite frequent and that mini chromosomes may be mobile.

As described above, Peng and colleagues found mini chromosomes of approx. 2 mb in the *M. oryzae* strain B71 carrying potential effector genes [3]. Interestingly, some sequences on the mini chromosomes were derived or duplicated from the ends of core chromosomes (from chromosomes 3, 6, and 7), including the effector genes PWL2 and BAS1. Furthermore, the analysis of repetitive sequences from the mini chromosomes showed that the transposons found on mini chromosomes are also enriched at the ends of core chromosomes. The implication of these findings is that sequences on mini chromosomes may in fact be mobile.

The second observation concerning the speed at which mini chromosomes can change, comes from studies in which changes in size and number of mini chromosomes were observed immediately after sexual or parasexual events [14,29,36,39,70,78]. The common conclusion of these studies is that these elements can change very quickly.

### 3.3. Why Are Effector Genes Abounding on Mini Chromosomes?

It is commonly accepted that resistance due to the recognition of avirulence factors by R gene products can be broken by the loss of the effector gene leading to a new round of adaptation [90]. With regard to a host jump, it was suggested that such an effector loss may be particularly important for large host jumps as the potential targets of the lost virulence factors may be missing in the naïve, new host [91]. Having several virulence determinants on a single mini chromosome may consequently confer an additional selective advantage, as several potential avirulence genes can be lost collectively as part of the same mini chromosome. This is also an advantage over lineage-specific accessory regions integrated into subtelomeric regions because those cannot be lost easily. For these reasons, mobility of genes between subtelomeric regions and mini chromosomes may be occurring quite regularly. Changing their chromosomal context may also have a strong effect on their expression, for example by mechanisms like telomere silencing [92]. Another interesting question to be asked about the ecology of mini chromosomes is why they are often—though not only—found in fungi blocked in the haploid stage. Meiosis is dependent on continuous stretches of homology as unsynapsed chromosomes activate meiotic checkpoints [93]. This should lead to a continuous selection against the formation of genomic rearrangements or the generation of novel mini chromosomes. Such a mechanism was suggested by Kistler and Miao [94] who used the term meiotic maintenance. They argued for the absence of accessory chromosomes in those fungi where meiosis is an essential part of the life cycle, like *Ustilago maydis*. This may be one of the reasons why they are often found in fungal lineages, such as *Colletotrichum*. In such a scenario, mini chromosomes may not only contribute to fast-evolving effector repertoires but also to the genetic isolation observed in the many species within the *Colletotrichum* genus and finally may also contribute to speciation [95]. 

While we now have very good evidence for the biological importance of mini chromosomes in virulence, many of the important mechanistical questions concerning the mechanisms of their generation and variation can only be answered as we start obtaining more whole-genome assemblies of this class of exciting pathogens.

## Figures and Tables

**Table 1:** Filamentous ascomycetes harboring mini chromosomes.

Species ^1^	Number	Size	Methods	Role in Virulence	Reference
***C. higginsianum*** (clade *destructivum*)	2	0.6 mb to 0.8 mb	PacBio genome assembly, optical mapping, PFGE	yes	[20,21,22]
***C. lentis*** (clade *destructivum*)	2	0.3 mb to 1.3 mb	Sequencing, optical mapping	yes	[23]
***C. graminicola*** (clade *graminicola)*	3	0.51 mb to 0.76 mb	Sequencing, optical mapping	no evidence	[22]
***C. acutatum*** (clade *acutatum*)	1 to 2	0.1 mb to 1 mb	PFGE	no evidence	[24]
***C. gloeosporioides*** (clade *gloeosporioides*)	2 to 11	0.29 mb to 2 mb	PFGE	possibly	[11,13,14,25]
***C. kahawae*** (clade *gloeosporioides*)	2 to 5	0.25 mb to 1.2 mb	PFGE, RAD sequencing ^2^	yes	[26]
***C. lindemuthianum*** (clade *orbiculare*)	2 to 6	0.5 mb to 2 mb	PFGE	no evidence	[27]
***Fusarium oxysporum***	1 to 2	1 mb to 2 mb	PFGE, sequencing	yes	[28,29]
***Magnaporthe oryzae***	1 to 2	1 mb to 3 mb	PFGE, southern blot, sequencing	yes	[3,30]
***Botrytis cinerea***	1 to 3	0.22 mb to 0.58	PFGE, sequencing	no evidence	[31,32]
***Alternaria alternata***	1	1 mb to 2 mb	PFGE, southern blot	yes	[33,34]
***Leptospharia maculans***	1	0.65 mb to 1 mb	PFGE, sequencing	yes	[35,36]
***Zymoseptoria tritici***	up to 8	0.41 mb to 0.77 mb	PFGE, genetic mapping	no evidence	[37,38,39]

^1^ Sorted by phylogenetic relation to *C. higginsianum* based on [40,22]. Pulsed-Field Gel Electrophoresis (PFGE). ^2^ Restriction site-assisted DNA sequencing.
